# Protective Effects of Sesamin on Cytoxan-Induced Spermatogenesis Dysfunction by Regulating RNF8-ubH2A/ubH2B Pathways in Male Mice

**DOI:** 10.3389/fphar.2021.708467

**Published:** 2021-09-13

**Authors:** Dong-Mei Hai, Jia-Wei Ren, Yan-Nan Chi, Rui-Juan Ye, Ning Liu, Lin Ma, Xiao-Bing Lan, Jing Wu, Jian-Qiang Yu, Jia-Mei Yang

**Affiliations:** ^1^ Department of Pharmacology, Ningxia Medical University, Yinchuan, China; ^2^ Laboratory Animal Center, Ningxia Medical University, Yinchuan, China; ^3^ Ningxia Hui Medicine Modern Engineering Research Center and Collaborative Innovation Center, Ningxia Medical University, Yinchuan, China

**Keywords:** sesamin, cytoxan (CTX), RNF8, ubH2A, ubH2B, spermatogenesis dysfunction

## Abstract

Most of the clinically infertile patients show spermatogenesis dysfunction. Cyclophosphamide, as an anticancer drug, can induce spermatogenesis dysfunction. Sesamin is the main bioactive component of natural lignans in sesame. It is abundant in sesame oil and has strong biological activities such as antioxidant, antibacterial, and hypoglycemic properties. By establishing the model of spermatogenic dysfunction induced by cyclophosphamide in male mice and then feeding sesamin (50, 100, and 200 mg/kg) for 2 weeks, we proved that sesamin can improve the reproductive organ damage induced by cyclophosphamide and increase the number and activity of sperms. Sesamin can resist cyclophosphamide-induced sperm nuclear maturity and DNA damage by increasing the expression levels of histones H2A and H2B in the testis. In addition, sesamin can improve the ubiquitination of histones regulated by RNF8 to protect the testis. In conclusion, these results suggest that sesamin can improve spermatogenic dysfunction induced by cyclophosphamide, which may be mediated by ubiquitination of histones.

## Introduction

Infertility is a reproductive dysfunction recognized by the clinical failure of pregnancy after 1 year or more of regular unprotected sexual intercourse (Zegers-Hochschild et al., 2009a). For the past 40 years, more than 50% of the total number and concentration of sperms has decreased in multiple countries up to date (Zegers-Hochschild et al., 2009b). It affects about 15% of couples of reproductive age, having failed pregnancy within a 12-month period under regular sexual intercourse (Punab et al., 2016). As a result, the reproductive health of infertile populations is also seen as a major public health concern worldwide.

Increasing research indicates that most of the infertile patients showed spermatogenesis dysfunction. However, the precise mechanisms underlying the spermatogenesis dysfunction in patients are still poorly elucidated. Moreover, the mechanisms influencing the spermatogenesis dysfunction are oxidative stress, inflammation, and direct cytotoxicity to spermatogenic cells as shown in numerous studies (Siu et al., 2009; Sunny O, 2013). Recently, accompanied by increasing epigenetic research on male infertility, the abnormal histone modification of the sperm nucleus during spermiogenesis has (Agarwal et al., 2017) become a major field of study. During the period of postmeiotic male germ cell development, histones are gradually replaced by protamine (Gou et al., 2017). Recent studies have shown that the ubiquitination of H2A (ubH2A) and H2B (ubH2B) regulated by the ubiquitin ligase RNF8 was a pivotal preliminary step in this process (Gou et al., 2017). Growing evidence collected in recent years indicates that abnormal histone modification can disrupt the spermiogenesis process and reduce semen quality. However, few clinically suitable drugs are currently available for spermatogenesis dysfunction treatment. Therefore, finding a treatment for spermatogenesis dysfunction and providing patients with several choices have become a major research hotspot.

Since ancient times, phytochemicals have been used in traditional Chinese medicine (TCM) for their health benefits. Recent studies have demonstrated the efficacy of TCM in male infertility, and it is expected to be a perfect candidate for pharmaceutical drug design (Zhou et al., 2015). Sesamin, a lignan isolated from the seeds of *S. indicum* plants and sesame oil, has several important biological properties, including antioxidant and anti-inflammatory activities (Zhou et al., 2015). Preclinical investigations have demonstrated that sesame and sesame oil exhibit potent effects on male factor infertility, and the clinical potential of these compounds has also been highlighted by other studies, further substantiating their role as fertility agents (Ashamu et al., 2010; Abbasi et al., 2013; Khani et al., 2013).

## Materials and Methods

### Experimental Animals

All animals received humane care in accordance with the “Guide for the Care and Use of Laboratory Animals” prepared by the National Academy of Sciences and published by the National Institutes of Health. A total of 120 healthy young-adult male mice (22–26 g) grown in the Institute of Cancer Research (ICR) were provided by the Experimental Animal Center of Ningxia Medical University (certificate number was SCXK Ningxia 2020-0001). The animals were allowed access to chow and water ad libitum and maintained at 23°C with a 12/12 h light–dark cycle.

### Drug Administration and Experimental Design

Sesamin (Shanghai Yuanye Bio-Technology Co., Ltd., Shanghai, China; CAS, 607-80-7, purity 98%), saline solution (vehicle, Beijing Solarbio Biotechnology Co., Ltd., Beijing, China), and sodium carboxymethyl cellulose (Beijing Solarbio Science and Technology Co., Ltd., Beijing, China; CAS, 9004-32-4) were used. All mice were randomly divided into five groups (twenty per group) after acclimatization for 4 days: the control group (standard chow plus vehicle *via* gavage), the CTX-treated group (standard chow plus 120 mg/kg/week cyclophosphamide dissolved in normal saline *via* gavage), and the CTX plus sesamin (50 mg/kg, 100 mg/kg, and 200 mg/kg sesamin dissolved in 0.5% CMC with chow plus cyclophosphamide *via* gavage) group.

### Sample Processing

After 2 weeks of sesamin treatment, all of the mice were sacrificed. Each mouse was dissected. The testicular tissues were divided into two samples. One sample was collected in the paraffin blocks after they were fixed in an animal testicular-tissue fixation fluid (Wuhan Servicebio Biotechnology Co., Ltd., Wuhan, China, Cat, G1121-500ML) for 24 h. Sections of 5 μm were obtained, deparaffinized, and stained with hematoxylin and eosin (H&E). The other sample was frozen at −80°C for subsequent biochemical analysis experiments, including assay kits and western blot analysis.

### Body Weight and Reproductive Organ Weight

Two testis tissues and epididymis were removed and weighed individually. Moreover, the reproductive organ coefficients were calculated.

### Semen Analysis

The mice were sacrificed at the end of treatment. The left cauda epididymis was put into a 2 ml saline solution at 37°C and simultaneously cut into small pieces for an entire sperm releasing at a sustaining temperature of 37°C for 5 min. Filtered with a 200-mesh cell sieve, then ix the sperm suspension and sperm fixative were mixed in a 1:4 ratio. The amount of spermatozoa and the percentage of motile spermatozoa were recorded under a light microscope at 400× magnification within 5 min by using a Neubauer cell-counting chamber. The diluted sperm suspension was dropped on the slide, and then 0.5% gentian violet solution was added. The sperm deformity rate was observed under a light microscope (Ma et al., 2005).

### Aniline Blue Staining

The aniline blue dye (Jiangxi Gelatins Biotechnology Co., Ltd., Jiangxi, China) can specifically bind to histones to reflect the nuclear maturity of the sperm. The air-dried smears of semen were used in this staining. The prepared slides were fixed in a fixation fluid for 15 min. Subsequently, they were rinsed in water and stained with aniline blue (Jiangxi Gelatins Biotechnology Co., Ltd., Jiangxi, China) for 5 min, followed by rinsing in water for 5 min. Under a light microscope at 400× magnification, two different types of chromatin were observed, with light blue as normal and dark blue as abnormal (Pourentezari et al., 2016).

### Acridine Orange Test

The acridine orange test is a metachromatic fluorescence staining used to determine the rate of spermatozoa with DNA denaturation (Taghizabet et al., 2016). Acridine orange (Beijing Solarbio Science and Technology Co., Ltd., Beijing, China; CAS, 10127-02-3) was dissolved in distilled water at a concentration of 1 mg/ml. In total, 10 μl of this solution was placed on a preheated (about 70°C) cleaned glass slide, spread by moving a glass rod back and forth, and air-dried. The AO-coated glass slides were stored at room temperature. Then, 10 µl of sperm at a concentration of five million per milliliter was gently dropped onto the slide; the slide was covered using a cover slide and placed in a Petri dish with a layer of moist paper. The slides were kept for 2 h. Under a fluorescent microscope (Olympus, Tokyo, Japan) with a 460-nm and 530-nm filter, two different types of DNA denaturation were observed, with green fluorescence as normal and red fluorescence as abnormal (Bahmanzadeh et al., 2016).

### Histopathological Evaluation Using Light Microscopy

All fixed testis and epididymis samples were embedded in paraffin and sectioned into slides with a thickness of 5 μm. Then, the slides were stained by H&E, followed by a conventional protocol for morphological observation.

### Western Blot Analysis

The proteins in the testis samples were immediately extracted, and the protein concentrations were determined using the BCA assay kit (Vazyme Biotechnology, Nanjing, China). Proteins were denatured, separated on a 10% SDS-PAGE gel, and transferred onto a nitrocellulose membrane. After electrotransfer, the membranes were blocked in PBST containing 5% nonfat dry milk for 2 h and then incubated overnight at 4°C with the following antibodies: H2A (1:1,000, ab18255, Abcam, United Kingdom), H2B (1:10,000, ab1790, Abcam, United Kingdom), and RNF8 (1:800, 14112-1-AP, Proteintech, United States). After washing thrice with PBST, the membranes were incubated with the secondary antibody (anti-rabbit IgG, 1:2000, SA00001-2, Proteintech Group, United States) for 2 h at room temperature. The bands were visualized using the Pierce® ECL Western blot substrate (Thermo Fisher Scientific, United States). The signals were quantified using the Western blot detection system Quantity One 4.31 (Bio-Rad, United States).

### Immunofluorescence Assays

Immunohistochemistry: after baking and dewaxing, the tissue sections were put into the EDTA (Beijing Solarbio Science and Technology Co., Ltd., Beijing, China; CAS, 6381-92-6) buffer (1 mM, pH 6.0) and the antigen was extracted using a microwave oven. After blocking the nonspecific sites with goat serum (Beijing Zhongshan Jinqiao Biotechnology Co., Ltd., Beijing, China, ZLI-9022) at room temperature, the slides were incubated with primary antibodies ubH2A (1:500, ab193203, Abcam, United States) and ubH2B (1:1,600, #5546, Cell Signaling, United States) at 4°C overnight. Then, the tissue slides were incubated with the tetramethylrhodamine isothiocyanate–labeled secondary antibody for 1 h, and then DAPI (Beijing Zhongshan Jinqiao Biotechnology Co., Ltd., Beijing, China, ZLI-9557) staining was performed. The slides were covered and analyzed.

### Statistical Analysis

Statistical analysis was performed using SPSS 22.0 statistical software (IBM, United States). The data were statistically evaluated using the one-way analysis of variance. All values were expressed as the mean ± standard deviation. The significance between the groups was determined using Student’s paired *t*-test. The values were considered significant at *p* < 0.05.

## Results

### Effects of Sesamin on the Reproductive Organ Weight and Sperm Quality in Cytoxan-Exposed Mice

After 2 weeks of treatment, the reproductive organ weight of the CTX group was significantly lower than that of the control mice (*p* < 0.001). The reproductive organ weight of Sesamin-treated (100 mg/kg and 200 mg/kg) mice was significantly higher than that of the CTX-exposed mice (*p* < 0.05, *p* < 0.01, and *p* < 0.001, Table 1).

**TABLE 1:** Effect of sesamin on the reproductive organ coefficient in mice with male spermatogenic dysfunction at 2 weeks (n = 10, mean ± SEM).

Group	Organ coefficient = genital/body weight × 100		
	Testis	Epididymis	Seminal vesicle
Control	0.349 ± 0.008	0.121 ± 0.004	0.709 ± 0.043
CTX	0.298 ± 0.007***	0.103 ± 0.002***	0.573 ± 0.018***
CTX + sesamin 50 mg/kg	0.303 ± 0.06	0.108 ± 0.003	0.632 ± 0.036
CTX + sesamin 100 mg/kg	0.328 ± 0.005^#^	0.113 ± 0.004^#^	0.681 ± 0.038^#^
CTX + sesamin 200 mg/kg	0.345 ± 0.008^###^	0.120 ± 0.002^###^	0.691 ± 0.011^##^

The table shows the reproductive organ coefficient in the control and experimental groups. Each value indicates the mean ± standard error of the mean (n = 10). *p < 0.05, **p < 0.01, and ***p < 0.001 vs. the control group; ^#^ *p* < 0.05, ^##^ *p* < 0.01, and ^###^ *p* < 0.001 vs. the CTX group.

**FIGURE 1:**
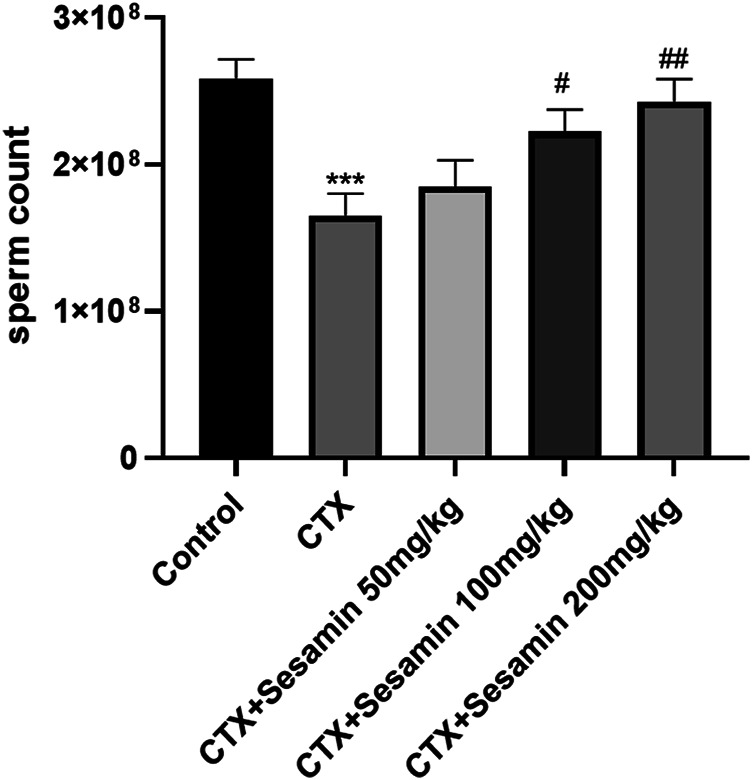
Sesamin attenuates sperm counts induced by CTX exposure in ICR mice. The results are presented as mean ± SEM (n = 10). *p < 0.05, **p < 0.01, and ***p < 0.001 as compared with the control group; ^#^ *p* < 0.05 and ^##^ *p* < 0.01 as compared with the CTX group.

**FIGURE 2:**
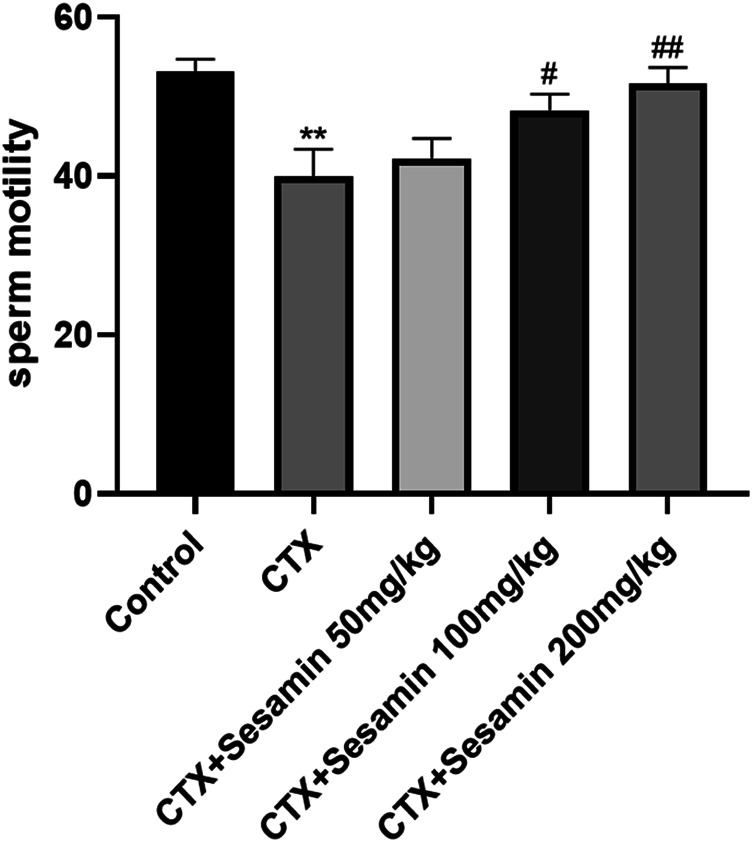
Sesamin attenuates sperm motility induced by CTX exposure in ICR mice. The results are presented as mean ± SEM (n = 10). *p < 0.05 and **p < 0.01 as compared with the control group; ^#^ *p* < 0.05 and ^##^ *p* < 0.01 as compared with the CTX group.

**FIGURE 3:**
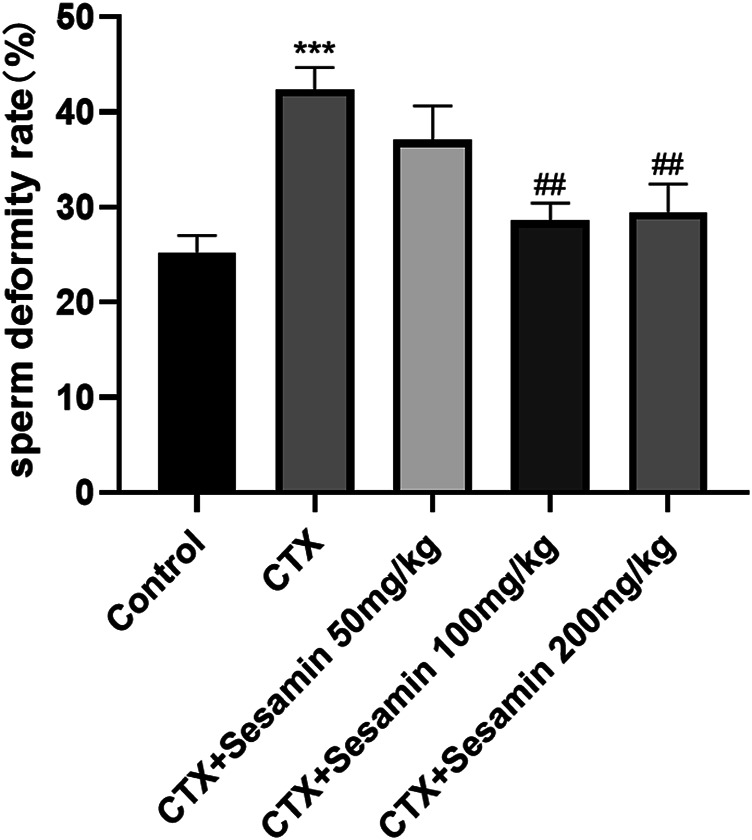
Sesamin attenuates the sperm deformity rate induced by CTX exposure in ICR mice. The results are presented as mean ± SEM (n = 6). ***p < 0.001 as compared with the control group; ^##^ *p* < 0.01 as compared with the CTX group.

After 2 weeks of treatment, the sperm concentrations and sperm motility of the CTX group were significantly lower than those of the control mice (*p* < 0.05, *p* < 0.001). The sperm concentrations and sperm motility of the sesamin-treated (100 mg/kg, 200 mg/kg) mice were significantly higher than those of the CTX-exposed mice (*p* < 0.05, *p* < 0.01). In addition, the sperm deformity rate of the CTX group was significantly higher than that of the control group (*p* < 0.001). The mice treated with sesamin (100 mg/kg and 200 mg/kg) showed significantly lower sperm deformity rate than CTX mice (*p* < 0.01, Figure 1, Figure 2, Figure 3).

### Effect of Sesamin on Testicular Morphological Damage Induced by Cytoxan

**FIGURE 4:**
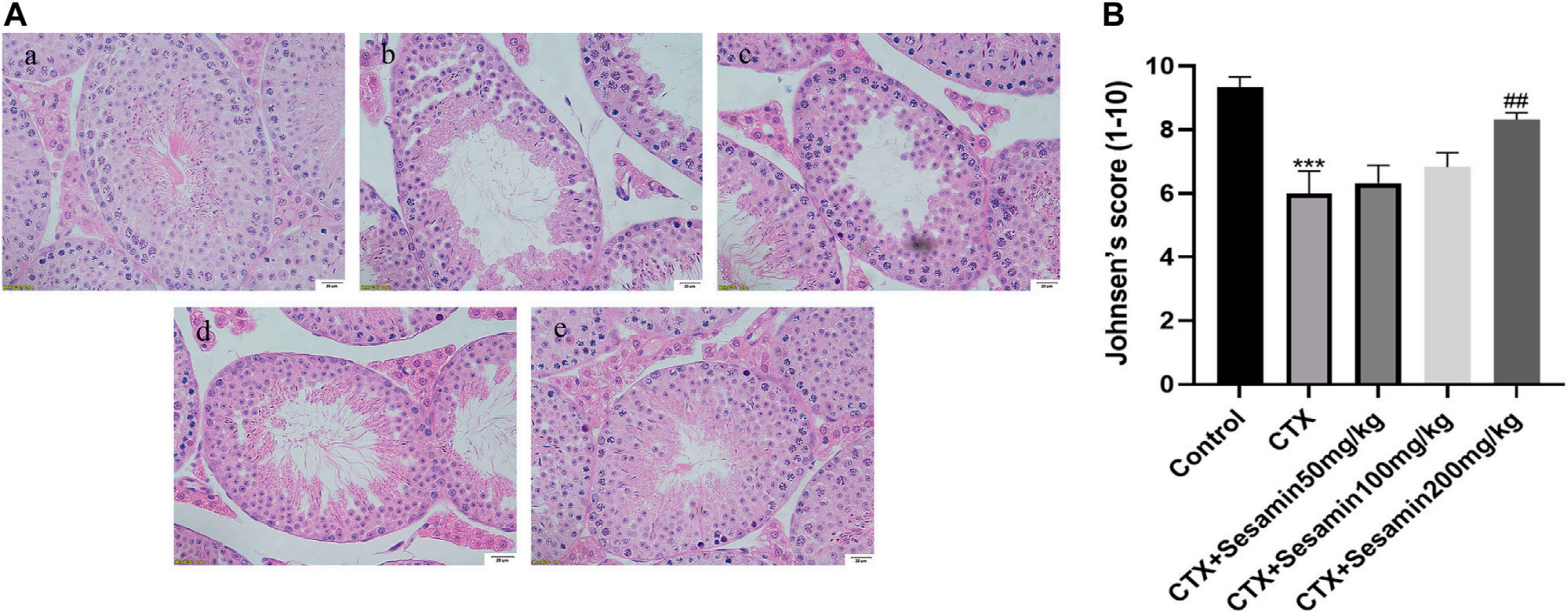
Effects of sesamin treatment on the CTX-induced male mice testicular histopathology. **(A)** The representative H&E staining photomicrographs in the seminiferous tubules of the testis at 400× magnification (scale bar = 20 μm). **(a)** Control group; **(b)** CTX group; **(c–e)** CTX + sesamin groups (50, 100, or 200 mg/kg). **(B)** Johnsen’s testicular score. Each bar indicates the mean ± standard error (n = 6). ***p < 0.001 vs. the control group; ^##^ *p* < 0.01 vs. the CTX group.

Under an optical microscope, seminiferous tubules of the control group displayed 5–7 layers of spermatogenic cells, which were orderly and closely arranged in the seminiferous tubules; at the same time, we can observe a lot of mature sperms in the lumen (Figure 4Aa). In the CTX group, spermatogenic cells were arranged in irregular spaces and the layer of spermatogenic cells was significantly reduced (Figure 4Ab). Compared with the control group, Johnsen’s score of the CTX group was significantly lower (*p* < 0.001, Figure 4B). The morphology of the testicular tissue in the sesamin (200 mg/kg) group was better than that in the CTX group, the spermatogenic cell layer increased significantly, and the spermatogenic cells were arranged orderly and tightly. There were more mature sperms in the lumen (Figure 4Ae), and Johnsen’s score of the sesamin group was significantly higher than that in the CTX group (*p* < 0.01, Figure 4B). These results indicate that CTX exposure can damage testicular tissue of mice, and sesamin can reverse this damage.

### Effects of Sesamin on Cytoxan-Induced Spermiogenesis Damage

**FIGURE 5:**
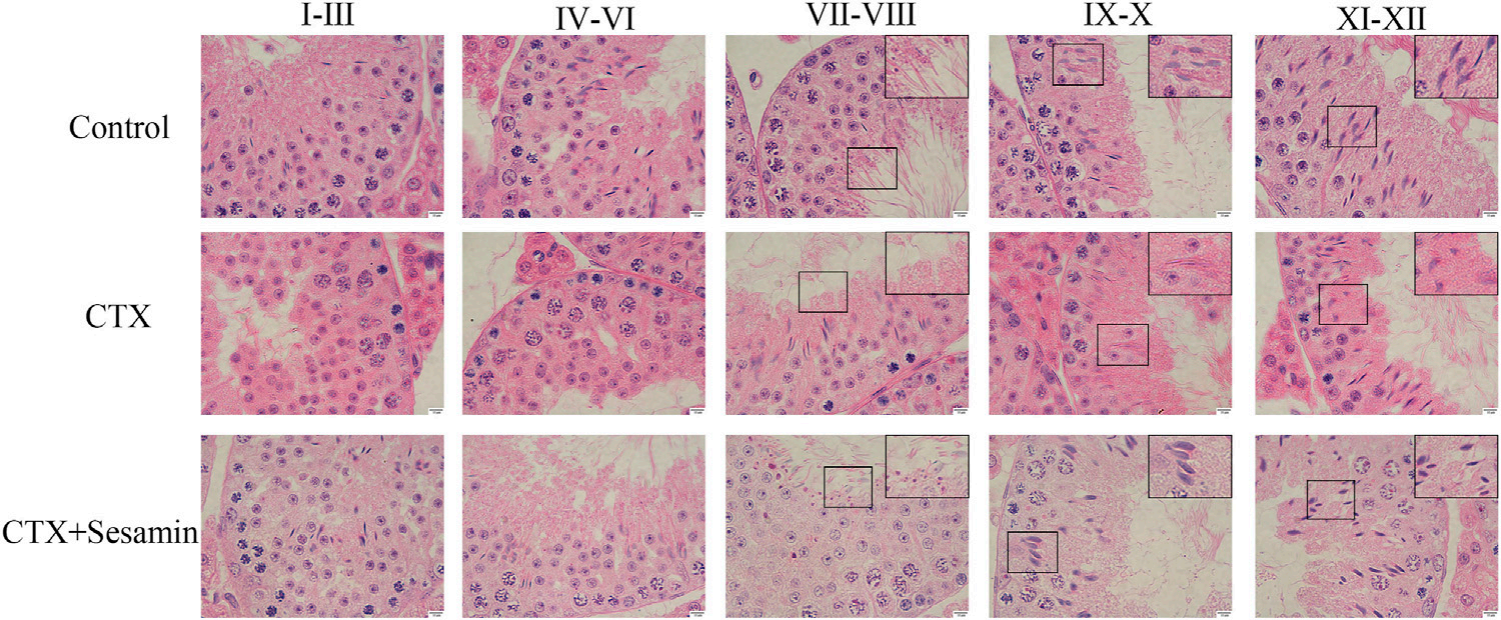
Stages of seminiferous epithelium cycles determined by H&E staining. The representative H&E staining photomicrographs in the seminiferous epithelium cycles of the testis at 1,000× magnification (scale bar = 10 μm).

Next, we analyzed the postmeiotic development of the testis in CTX mice. The spermatogenic epithelial cycle is roughly divided into five stages (Jie et al., 2006). In stages I–III, spermatocytes of the early pachytene stage are located near the basal surface, with small cells and round nuclei. Chromatins are compact and punctate or cordlike, occupying most of the nuclei. In stages IV–VI, type B spermatogonial cells can be seen partly in contact with the basal surface, the nuclei of which are oval, as well as proline spermatogonial cells differentiated from type B spermatogonial cells. The cells and nuclei are small, and the nuclei are deeply stained, located on the basal surface of pachytene-stage spermatogonial cells. Release of mature spermatozoa into the lumen occurs at stages VII–VIII. The spermatids in the deformation phase were observed in the stages IX–X, and the deformed spermatozoa were long and fusiform. There were more spermatids in the stages XI–XII, but no round spermatids. We found that, in the CTX group, the number and morphology of spermatogenic cells at all levels in the testis did not change during the early stages of spermatogenesis, I–VI, while the number of mature spermatogenic cells entering the lumen decreased during the stages VII–VIII and the number of spermatogenic cells decreased during the deformable stages IX–XII, indicating that CTX mainly affected the late stage of spermatogenesis. Compared with the CTX group, the number of mature spermatogenic cells and deformed spermatozoa increased after treatment with sesamin 200 mg/kg (Figure 5).

### Effects of Sesamin on Sperm Nuclear Damage Caused by Cytoxan

#### Acridine Orange Staining

**FIGURE 6:**
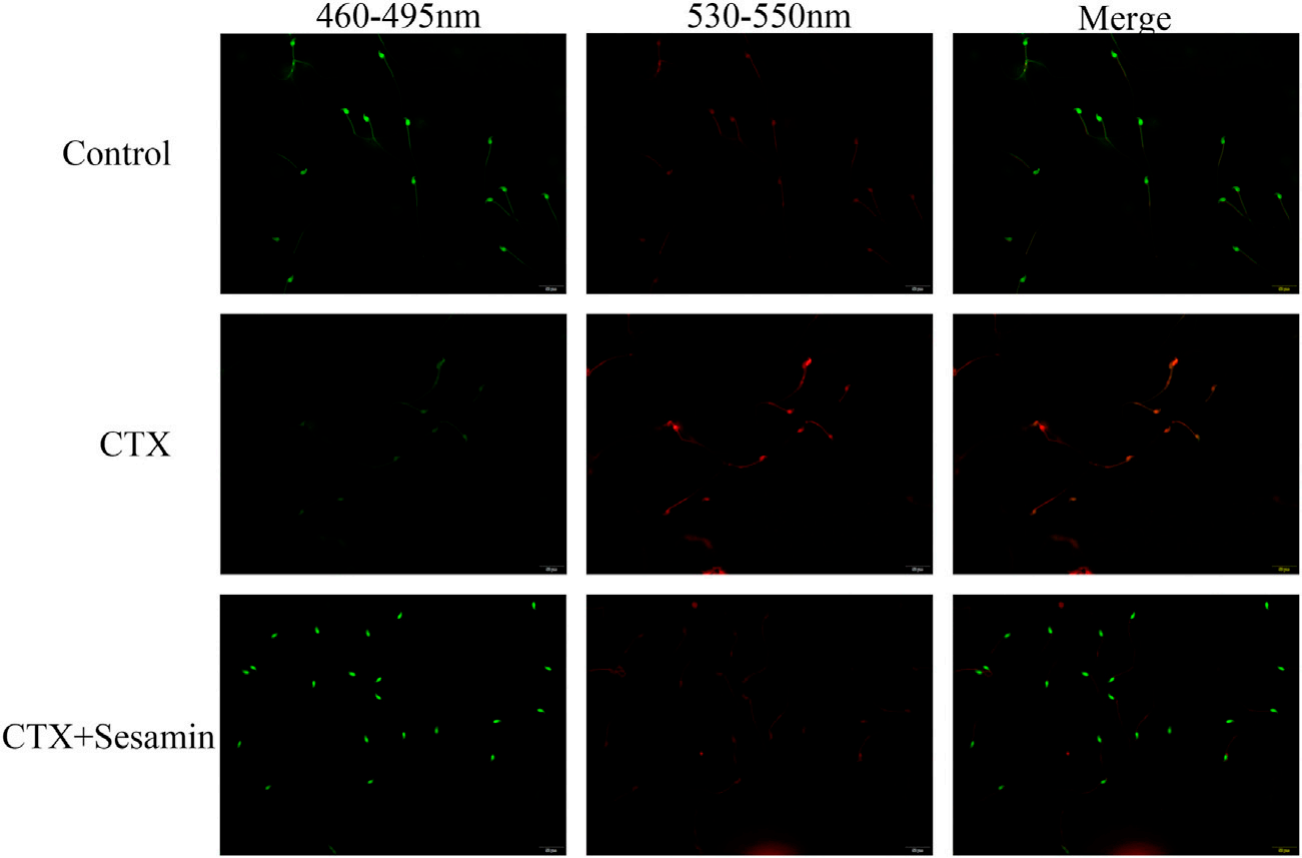
Typical results of acridine orange on sperm smears. The green signal indicates spermatozoa which contain double-stranded DNA, while orange and red signals imply spermatozoa with single-stranded DNA or RNA.

Acridine orange staining was used to determine the capability of nuclear DNA of spermatozoa in denaturation. The spermatozoon head with non-denatured DNA is green, and denatured DNA has orange-red color. Compared with the control group, the nuclear DNA of spermatozoa in the cyclophosphamide group was damaged and sesame seeds reversed this damage (Figure 6).

#### Toluidine Blue Staining

**FIGURE 7:**
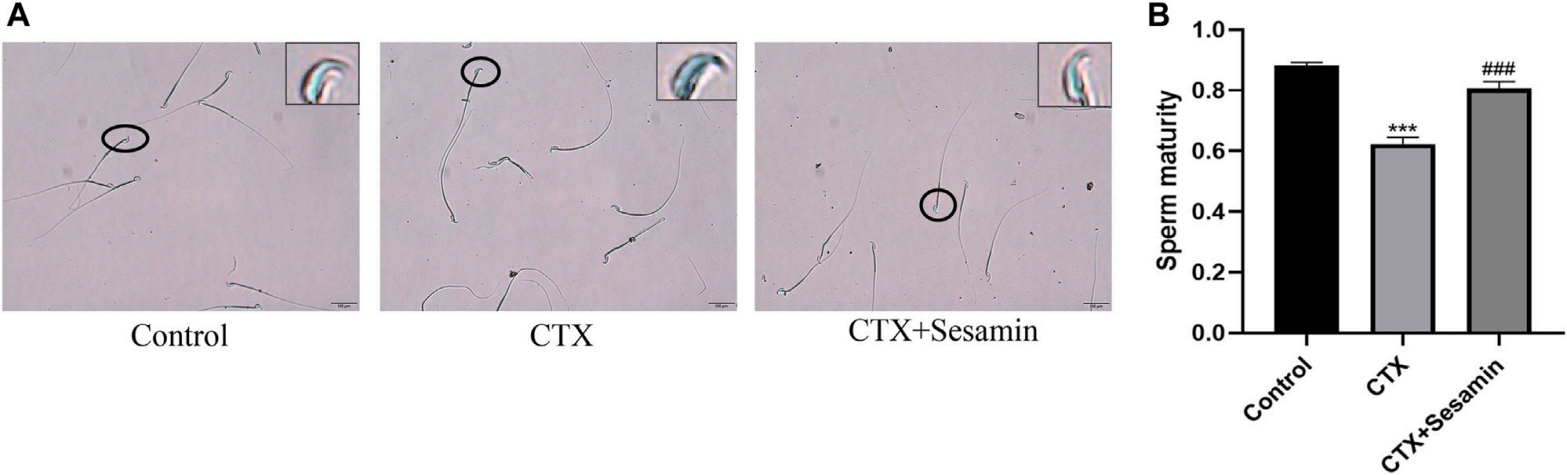
Typical results of toluidine blue on sperm smears. **(A)** Toluidine blue staining shows the chromatin structure of the sperm. Light blue indicates the sperm nucleus with a good chromatin structure, and dark blue indicates an abnormal chromatin structure. **(B)** Sesamin attenuates sperm chromatin condensation induced by CTX exposure in ICR mice. The results are presented as mean ± SEM (n = 6). ***p < 0.001 as compared with the control group; ^###^ *p* < 0.001 as compared with the CTX group.

This staining shows the amount of sperm chromatin condensation. Toluidine blue staining shows the sperm chromatin structure. Light blue indicates the sperm nucleus with a good chromatin structure, and dark blue indicates an abnormal chromatin structure (Figure 7A). Figure 7B shows that CTX increased the mean percentage of abnormal chromatins compared to the control. Sesamin (200 mg/kg groups) decreased the abnormal chromatin condensation compared with the CTX group (*p* < 0.001).

### Effects of Sesamin on RNF8, H2A, and H2B Protein Expressions in the Testicular Tissue

**FIGURE 8:**
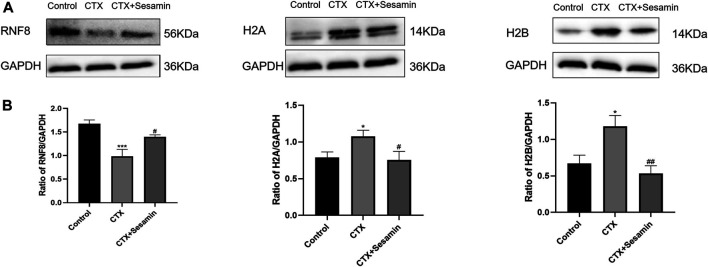
Effects of sesamin (200 mg/kg) on the expression of RNF8, H2A, and H2B in the testicular tissue. **(A)** The representative western blot analysis of RNF8, H2A, and H2B activation in the testicular tissue for the different groups. **(B)** Statistical analysis of the gray value of RNF8, H2A, and H2B for the different groups. The data indicate the mean ± standard error (n = 6). ***p < 0.001 vs. the control group; ^#^ *p* < 0.05 vs. the CTX group.

To investigate the increase RNF8, H2A and H2B expressions in testis tissue of the mice treated sesamin, Western blot analysis was conducted. As shown in Figure 8, RNF8 was repressed after CTX exposure (*p* < 0.001), and sesamin (200 mg/kg) notably stimulated the upregulation of it to a moderate level (p < 0.05). The H2A and H2B protein levels in the CTX group were markedly increased compared with the control group (*p* < 0.05). The administration of sesamin (200 mg/kg) to CTX mice produced a significant decrease in RNF8 protein expressions (*p* < 0.05, *p* < 0.01, Figure 8).

### Effects of Sesamin on ubH2A and ubH2B Immunoreactivities in the Testis of Cytoxan-Induced Mice

**FIGURE 9:**
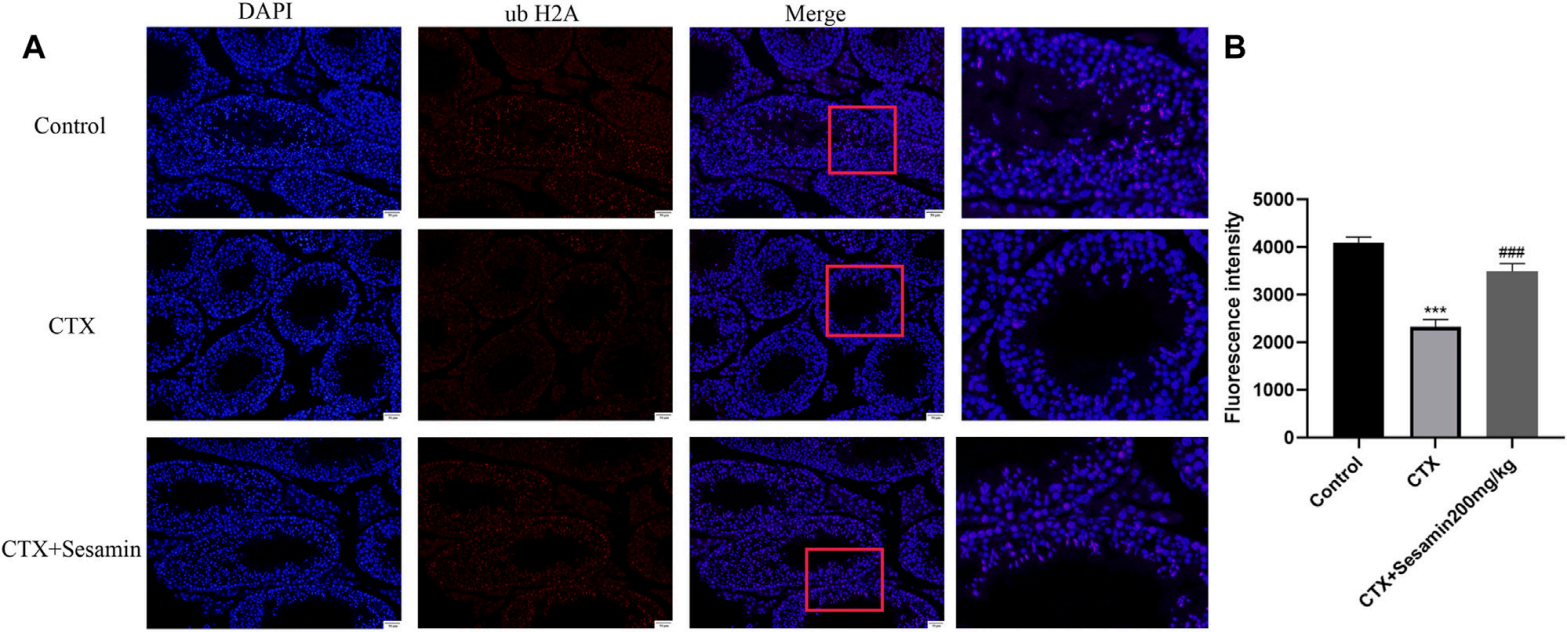
The expression of ubiquitin histone H2A in the testis. The representative fluorescence photomicrographs in the seminiferous epithelium cycles of the testis at 200× magnification (scale bar = 50 μm). **(A)** The immunofluorescence analysis of ubiquitin histone H2A in the seminiferous tubule at stages Ⅺ–Ⅻ. **(B)** Statistical analysis of the fluorescence intensity of ubiquitin histone H2A in the different groups. The data indicate the mean ± standard error (n = 6). ***p < 0.001 vs. the control group; ^###^ *p* < 0.001 vs. the CTX group.

**FIGURE 10:**
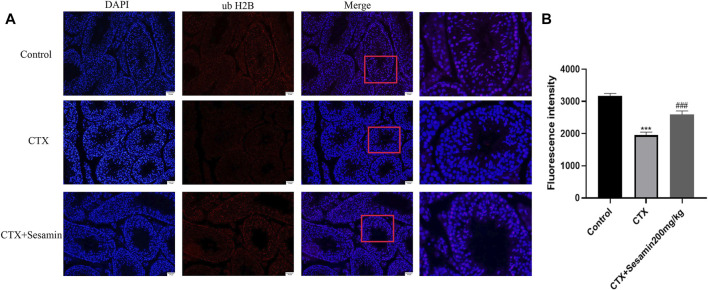
The expression of ubiquitin histone H2B in the testis. The representative fluorescence photomicrographs of the seminiferous epithelium cycles of the testis at 200× magnification (scale bar = 50 μm). **(A)** The immunofluorescence analysis of ubiquitin histone H2B in the seminiferous tubule at stages Ⅺ–Ⅻ. **(B)** Statistical analysis of the fluorescence intensity of ubiquitin histone H2B in the different groups. The data indicate the mean ± standard error (n = 6). ***p < 0.001 vs. the control group; ^###^ *p* < 0.001 vs. the CTX group.

Immunofluorescence analysis showed the number of positive cells of ub-H2A and ub-H2B (Figure 9A and Figure 10A). The fluorescence intensity of ubH2A and ubH2B was stronger in the control group (Figure 9B, and Figure 10B), while the expression of ubH2A and ubH2B was weaker in the CTX group (*p* < 0.001). However, sesamin (200 mg/kg) significantly increased the fluorescence intensity of ubH2A and ubH2B in the testis in the CTX group (*p* < 0.001).

## Discussion

Nowadays, the defective histone-to-protamine transition has been a key factor contributing to male sterility (Oliva, 2006; Yan, 2009). Also, there is no effective drug targeting this defect to ameliorate male infertility. Sesamin is the main natural lignolipid bioactive component in sesame, which exists in large quantities in sesame oil and has strong bioactive effects such as antioxidant, anti-inflammatory, and hypoglycemic activities (Chen et al., 2015; Ahmad et al., 2016; Guo et al., 2016; Majdalawieh et al., 2017). Sesamin commonly induces hormetic dose responses in numerous biological models for endpoints of biomedical and clinical relevance. For example, sesamin can prevent neurodegeneration related to the accumulation of oxidative stress by regulating the Nrf2-ARE pathway (Calabrese et al., 1996; Le and Inoue, 2021). Sesamin can inhibit the proliferation and invasion of cancer cells and can be used as a drug to prevent and treat cancer (Mottaghi and Abbaszadeh, 2021). Previous studies have shown that sesame, sesame alcohol extract, and sesame oil have protective effects on male fertility, which is closely related to their antioxidant activities. For instance, sesame can effectively improve male semen parameters because of its antioxidant properties, which is a safe and effective method for the treatment of male spermatogenic dysfunction (Khani et al., 2013). The vitamin C and ethanol extract of sesame can improve the fertility of rats through antioxidant effect (Ashamu et al., 2010). Other studies have shown that sesame oil can improve germ cells and support cells of diabetic rats to varying degrees and can dose-dependently increase the plasma testosterone concentration of diabetic rats (Abbasi et al., 2013). However, the effect of sesamin on male spermatogenic dysfunction has not been reported. This study first reported the potential beneficial effect of sesamin on CTX-induced mice with spermatogenesis dysfunction and explored the possible underlying mechanisms about the histone-to-protamine transition.

As have been shown previously, CTX mice had remarkably damaged testicular morphology, including disordered spermatogenic cells, inhibited spermatogenesis, and prominent morphological injuries to the Sertoli cells and spermatogonia (Chen and Wang, 2015; Huang et al., 2016). The macroscopic and microscopic changes of the testis in the CTX group were the same as previously reported, including significantly decreased testicular organ coefficient, irregular arrangement of spermatogenic cells, and significantly reduced number of layers of spermatogenic cells. However, in the sesamin-treated group (100 or 200 mg/kg), these histopathological changes showed different degrees of recovery. Morphological evidence showed that sesamin could significantly reduce testicular injury induced by CTX.

In all animals treated with CTX, the decrease in sperm count indicated the damaging effect of cyclophosphamide on the testicular germ cell cycle (Shittu et al., 2019). In this study, we found that after exposure to CTX, the concentration and motility of sperm decreased significantly and the rate of sperm deformity increased significantly, which is consistent with previous reports. The results showed that CTX could induce sperm damage in mice. In addition, compared with the CTX group, sesamin can increase the number and motility of sperm and reduce sperm abnormality rate. These results indicated that sesamin could improve CTX-induced sperm damage and had protective effect on the sperm. In order to understand the specific underlying mechanism, we examined each stage of the spermatogenic epithelial cell cycle by H&E staining, which showed that spermatogenesis was blocked during spermatogenesis and the number of concentrated spermatids in seminiferous tubules of CTX-exposed mice was greatly reduced, which was recovered by sesamin treatment.

Spermatogenesis is accompanied by the indispensable transformation of postmeiotic haploid sperm cells to mature elongated sperm cells, divided into 12 steps, involving the unique characteristics of histone-to-protamine exchange in the spermatozoon head. Chromatin recombination occurs uniformly during spermatozoon elongation (Li et al., 2019). Histones include five types, H1, H2A, H2B, H3, and H4, which together with DNA constitute the basic unit of chromosomes (Sheng et al., 2014). Current studies mostly focus on H2A and H2B. Therefore, we studied the sperm nuclear maturity, sperm DNA integrity, and expression of histones H2A and H2B in the testis. The results showed that after treatment with CTX, sperm nuclear maturity and sperm DNA integrity were destroyed and the expression levels of H2A and H2B in the testis were increased, suggesting that CTX exposure damaged sperm nuclear maturity and sperm DNA integrity and disrupted the histone–protamine exchange process. After sesamin treatment, the destruction of sperm nuclear maturity and DNA integrity caused by CTX was improved and the expression levels of H2A and H2B in the testis were significantly decreased. Since histone–protamine exchange is a prerequisite for the production of mature sperm, these results suggest that the improvement of incomplete histone–protamine exchange may be the basis of sesamin in the treatment of CTX-induced spermatogenic dysfunction. Histone ubiquitination promotes histone clearance during histone replacement by protamine. Recent studies have shown that ub-H2A and ub-H2B regulated by histone ubiquitin ligase RNF8 is a key step in histone removal (Lu et al., 2010; Gou et al., 2017). Therefore, we further studied the expression changes of ubH2A and ubH2B and the expression level of RNF8 in the testis. The results showed that the expression of ubH2A and ubH2B in the testis decreased significantly after treatment with CTX. Meanwhile, the expression level of RNF8 in the testis was also significantly decreased. These results indicate that exposure to cyclophosphamide disrupts histone ubiquitination. After sesamin treatment, the expression levels of ubH2A, ubH2B and RNF8 in the testis tissue were significantly increased compared with the model group and the histone ubiquitination was protected. Therefore, in this study, we believe that sesamin may induce complete exchange of histone and protamine in the sperm head through upregulation of RNF8-mediated histone ubiquitination, thus alleviating CTX-induced spermatogenesis dysfunction.

Rattan first put forward the term “vitagene” in 1998 to describe various maintenance and repair processes in cells (Rattan, 1998). The gene network operates on four levels, namely, molecular level, cell level, tissue and organ level, and physiological and redox control level. The molecular level is related to the antioxidant defense system, including DNA repair system, synthesis of stress protein, and proteasome degradation of damaged protein (Surai et al., 2019). In numerous experimental models, natural antioxidants central to TCM approaches and rationals (Wang et al., 2018) have shown to induce hormetic dose responses. Sesamin is a natural polyphenol antioxidant. Sesamin commonly induces hormetic dose responses in numerous biological models as endpoints of biomedical and clinical relevance. For example, sesamin can prevent neurodegeneration related to the accumulation of oxidative stress by regulating the Nrf2-ARE pathway (Fusco et al., 2021; Le and Inoue, 2021). The Nrf2-ARE axis plays a key role as an important signal to protect cells from endogenous and exogenous oxidative stress (de Freitas Silva et al., 2018). In various diseases related to neurodegeneration, chronic inflammation, aging, and cancer, this signaling is considered to be an essential defense mechanism against the harmful effects of oxidative stress (Jantan et al., 2015; Kageyama et al., 2014; Inoue et al., 2015; Tong et al., 2015; Li et al., 2018; Siracusa et al., 2020). In this study, sesamin can regulate RNF8-ubH2A/ubH2B to repair CTX-induced sperm DNA damage in male mice, which may also be related to its antioxidant properties. In conclusion, sesamin can protect against various diseases caused by oxidative stress by regulating Nrf2. Meanwhile, sesamin can repair DNA damage and protect spermatogenesis. Therefore, sesamin can be used as vitamin-regulating nutritional supplements, which has a potential public health and clinical significance.

## Conclusion

In this study, CTX exposure significantly reduced sperm count and motility in male mice. Interestingly, sesamin can effectively protect spermatogenesis of male mice from CTX-induced damage by normalizing histone modification and restoring histone–protamine exchange during spermatogenesis. In conclusion, the results of this study suggest that sesamin can prevent CTX-induced male reproductive dysfunction. This finding may provide new evidence and clues for understanding the protective mechanism of sesamin on CTX-induced reproductive toxicity. At the same time, it provides more evidence for sesamin to be used as a vitamin-regulating nutritional supplement.

## Data Availability

The original contributions presented in the study are included in the article/Supplementary Material; further inquiries can be directed to the corresponding authors.
